# Drying sea buckthorn berries (*Hippophae rhamnoides* L.): Effects of different drying methods on drying kinetics, physicochemical properties, and microstructure

**DOI:** 10.3389/fnut.2023.1106009

**Published:** 2023-02-08

**Authors:** Zhihua Geng, Lichun Zhu, Jun Wang, Xianlong Yu, Mengqing Li, Wenxin Yang, Bin Hu, Qian Zhang, Xuhai Yang

**Affiliations:** ^1^College of Mechanical and Electrical Engineering, Shihezi University, Shihezi, China; ^2^College of Food Science and Engineering, Northwest A&F University, Yangling, China; ^3^Shandong Academy of Agricultural Machinery Sciences, Jinan, China; ^4^Xinjiang Production and Construction Corps, Key Laboratory of Modern Agricultural Machinery, Shihezi, China; ^5^Engineering Research Center for Production Mechanization of Oasis Special Economic Crop, Ministry of Education, Shihezi, China

**Keywords:** drying kinetics, physicochemical properties, pulsed vacuum drying, vacuum freeze-drying, sea buckthorn berries

## Abstract

Sea buckthorn berries are important ingredients in Chinese medicine and food processing; however, their high moisture content can reduce their shelf life. Effective drying is crucial for extending their shelf life. In the present study, we investigated the effects of hot-air drying (HAD), infrared drying (IRD), infrared-assisted hot-air drying (IR-HAD), pulsed-vacuum drying (PVD), and vacuum freeze-drying (VFD) on the drying kinetics, microstructure, physicochemical properties (color, non-enzyme browning index, and rehydration ratio), and total phenol, total flavonoid, and ascorbic acid contents of sea buckthorn berries. The results showed that the IR-HAD time was the shortest, followed by the HAD, IRD, and PVD times, whereas the VFD time was the longest. The value of the color parameter L* decreased from 53.44 in fresh sea buckthorn berries to 44.18 (VFD), 42.60 (PVD), 37.58 (IRD), 36.39 (HAD), and 36.00 (IR-HAD) in dried berries. The browning index also showed the same trend as the color change. Vacuum freeze-dried berries had the lowest browning index (0.24 Abs/g d.m.) followed by that of pulsed-vacuum–(0.28 Abs/g d.m.), infrared- (0.35 Abs/g d.m.), hot-air–(0.42 Abs/g d.m.), and infrared-assisted hot-air–dried berries (0.59 Abs/g d.m.). The ascorbic acid content of sea buckthorn berries decreased by 45.39, 53.81, 74.23, 77.09, and 79.93% after VFD, PVD, IRD, IR-HAD, and HAD, respectively. The vacuum freeze-dried and pulsed-vacuum–dried sea buckthorn berries had better physicochemical properties than those dried by HAD, IRD, and IR-HAD. Overall, VFD and PVD had the highest ascorbic acid and total phenolic contents, good rehydration ability, and bright color. Nonetheless, considering the high cost of VFD, we suggest that PVD is an optimal drying technology for sea buckthorn berries, with the potential for industrial application.

## 1. Introduction

Sea buckthorn (*Hippophae rhamnoides* L.), of the family Hojicaceae (1) is a perennial deciduous shrub and a berry fruit-bearing tree. This species is light-loving, cold- and heat-tolerant, and can survive in sandy and arid climates in saline soils. Consequently, it is widely used for soil and water conservation and desert greening. Sea buckthorn is a collective name for the plant and its fruits, which are used as food and have medicinal properties (2). Sea buckthorn is widely distributed and is found in temperate zones of Europe and Asia. It is mainly distributed in Eurasia, particularly in China, which is home to the most species. The area of sea buckthorn cultivation worldwide is 918,700 hm^2^ with annual production of 400,000 tons, and that in China is 780,900 hm^2^ with a production of annual production 250,000 tons (3).

Sea buckthorn berries are used as raw ingredients in Chinese medicine, food processing, and other industries. The berries are rich in flavonoids, vitamins, polyphenols, and other nutrients, and can promote blood circulation and disperse blood stasis, dissolve phlegm and broaden the chest, generate body fluid and quench thirst, and cure diarrhea (4). Sea buckthorn berries contain flavonoid compounds, mainly quercetin, kaempferol, and isorhamnetin, which possess anti-inflammatory activity, regulate the intestinal flora, and exert various physiological effects, including antioxidant release, blood glucose regulation, anti-inflammatory action, and hypolipidemia, and thus help prevent and aid in the treatment of heart diseases, hypertension, hyperlipidemia, hyperglycemia, and cancer (5). Flavonoid compounds in sea buckthorn berries (as nutritional supplements) help regulate intestinal microecology and a significant negative correlation exists between the intake of sea buckthorn berries and the incidence of metabolic diseases (6). Moreover, sea buckthorn berries are rich in vitamin C, have high stability, and find considerable application in the cosmetic industry (7). The vitamins in the berries also play an important role in scavenging free radicals, improving blood circulation, and anti-cellular differentiation. Nowak et al. (8) showed that ascorbic acid and polyphenols have a synergistic effect in determining the antioxidant properties of sea buckthorn juice and play an important role in disease prevention. Polyphenols in sea buckthorn berries are mainly phenolic acids such as protocatechuic acid, gallic acid, caffeic acid, p-coumaric acid, ferulic acid, and other major phenolic acids, and possess hypolipidemic, anticancer, and intestinal flora-improving effects (9). Their ability to inhibit or stimulate specific bacterial species to alleviate intestinal flora imbalance and reduce reactive oxygen species, inflammatory markers, harmful bacterial effects, and colonic tissue damage is important for maintaining intestinal health (10).

Fresh sea buckthorn berries contain up to 80% water and are rich in vitamins and polyphenols with unstable active ingredients that are easily oxidized, making them highly perishable commodities (11). Harvested berries are not suitable for long-term storage as they ferment and deteriorate in a short time at room temperature, thus losing their medicinal value. Therefore, extending the shelf life of sea buckthorn berries and reducing postharvest losses are important issues facing the sea buckthorn industry. In addition to methods such as pre-cooling and preservation, drying preserves the berries by reducing the water content in fruits and vegetables. Sea buckthorn berries are commonly dried by hot-air drying (HAD) through air convection and heat conduction for heat exchange (12). Although HAD is inexpensive and does not require specialized equipment, heat conduction is necessary for heat transfer. Vacuum freeze-drying (VFD) is an emerging technology that has been used to dry sea buckthorn berries in recent years (13). The color and shape of the berries remain largely unchanged after VFD, which minimizes quality deterioration, including enzymatic and non-enzymatic browning and protein denaturation, preserves nutrition, and maintains color (14). However, VFD equipment is expensive—approximately five times more expensive than that used in traditional drying methods—and the productivity is low, which limits the potential for wide application of this technology (15).

This study aimed to investigate the effects of different drying methods, namely HAD, infrared drying (IRD), infrared-assisted hot air-drying (IR-HAD), pulsed vacuum drying (PVD), and VFD, on the drying kinetics, physicochemical properties, and microstructure of sea buckthorn berries. The results could provide a basis for the selection of an optimal drying method.

## 2. Materials and methods

### 2.1. Materials

Fresh sea buckthorn berries were picked from No. 170 State Farm in E-ming (Tacheng, Xinjiang, China). The hand-selected sea buckthorn berries were of uniform size and color, with no bruises or rotting, and were stored at −20°C in a refrigerator. The average mass of the selected samples was 2.5 ± 0.1 g, with a long axis length of 50 ± 5 mm and a short axis length of 30 ± 5 mm. The surface dust was removed with water, and the remaining water was blotted off the surface using absorbent paper before the experiment. The average initial moisture content of sea buckthorn berries was measured by drying in a vacuum chamber at 70°C for 24 h (16).

### 2.2. Drying methods

In all tests, the dryer was first switched on and run for 30 min to obtain a steady state. The material (100 g) was first placed in a stainless-steel metal grid tray with a loading density of 0.2 kg/m^2^ and then placed in a drying chamber. The drying was stopped when the moisture content decreased below 10%. All drying experiments were conducted in triplicate. The results were averaged for analysis, and the material was sealed and packed after cooling.

**FIGURE 1:**
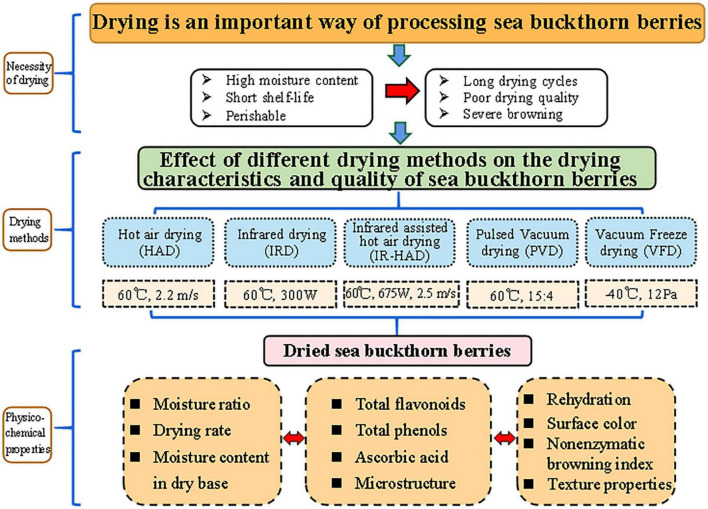
Schematic overview of the experimental process.

**FIGURE 2:**
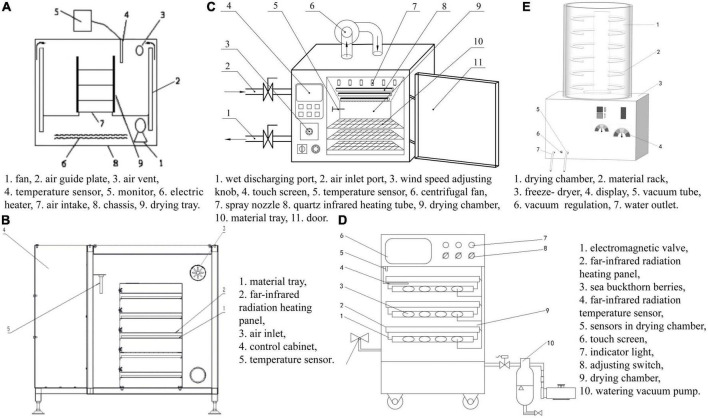
The schematics diagram of the five dryers. **(A)** Hot-air drying (HAD) [adapted from Zhang et al. (17)], **(B)** infrared drying (IRD) [adapted from Li (18)], **(C)** infrared-assisted hot-air drying (IR-HAD) [adapted from Chang et al. (19)], **(D)** pulsed-vacuum drying (PVD) [adapted from Deng et al. (16)], **(E)** vacuum freeze-drying (VFD) [adapted from Schössler et al. (20)].

Hot-air drying was performed as described by Zhang et al. (17). The HAD equipment was obtained from Shanghai Yihuan Scientific Instruments Co., Ltd. (Shanghai, China). The drying medium temperature was set at 60°C, the air speed was kept at 2.2 m/s, and the airflow direction was parallel to the drying material tray. IRD was performed as described by Li (18); the IRD equipment was custom-made from Shihezi University (Shihezi, China), with a power of 300 W and drying medium temperature of 60°C. IR-HAD was performed as described by Chang et al. (19). The IR-HAD equipment was obtained from Jiangsu Sentec Co., Ltd. (Shihezi, China), and drying medium temperature of 60°C, IR power of 675 W, and air speed of 2.5 m/s were used. Notably, IR radiation is the heat source in IR-HAD, and the IR radiation tube stops heating when the temperature reaches a set value, subsequently using HAD. When the temperature decreases again, the IR radiation tube starts heating once more. PVD was performed as described by Deng et al. (16). The PVD equipment was custom-made from Shihezi University (Shihezi, China). The thermal sensors were used to measure and control the far-infrared radiation heating panel temperature. The test was conducted at the far-infrared radiation heating panel temperature of 60°C, and the dryer was set with an atmospheric pressure holding time of 15 min and a vacuum holding time of 4 min. VFD was performed as described by Schössler et al. (20). HAD, IRD, and IR-HAD thermal sensors were used to measure and control the air temperature above the material tray. In the IR-HAD dryer, the distance between the infrared radiation tubes and the material tray was 120 mm. Three quartz glass medium-wave (2–4 μm) infrared tubes were placed parallel to the material tray, and their radiation power was 450, 225, and 225 W, respectively. During the drying experiment, two medium-wave infrared tubes, with a total radiation power of 625 W, were selected. The VFD equipment was obtained from CHRIST Freeze Dryer GmbH (Osterode, Germany), with a cold-trap temperature of −40°C, heating plate temperature of 30°C, and a drying chamber pressure of 12 Pa. Figure 1 shows the schematics of the five dryers, and Figure 2 shows the experimental setup process.

### 2.3. Drying characteristics

During drying, the variation of moisture content—moisture ratio (*MR*) and drying rate (*DR*)—sea buckthorn berries weight loss was measured every 30 min, and the MR-t and DR-MR curves were calculated (21).

The drying moisture ratio *MR* of sea buckthorn berries at drying time *t*, was obtained from Eq. 1:


(1) $$\mathrm{MR}=\frac{M_{t}-M_{e}}{M_{0}-M_{e}}$$

where *M*_0_ is the initial dry-basis moisture content in g/g; *M*_*e*_ is the dry-basis moisture content at the time of drying to equilibrium in g/g; *M*_*t*_ is the dry-basis moisture content at any drying *t* moment in g/g.

Because the equilibrium dry-basis moisture content *M*_*e*_ is much smaller than *M*_0_ and *M*_*t*_, Eq. 1 can be simplified to Eq. 2:


(2) $$\mathrm{MR}=\frac{M_{t}}{M_{0}}$$

The *DR* of sea buckthorn berries during the drying process can be derived from Eq. 3:


(3) $$\mathrm{DR}=\frac{M_{t\,1}-M_{t\,2}}{t_{1}-t_{2}}$$

### 2.4. Rehydration ratio

Based on the method of Zhang et al. (22), a glass with deionized water was placed in a water bath at 40°C. When the temperature of deionized water was constant, 5 g of dried sea buckthorn berries was added to the 50 mL deionized water. After 30 min, the berries were removed and drained, and the surface of the material was wiped dry with absorbent paper and weighed using an analytical balance after rehydration. The rehydration ratio was calculated according to Eq. 4:


(4) $$R_{r}=\frac{m_{2}}{m_{1}}$$

where *R*_*r*_ is the material rehydration ratio, g/g; *m*_2_ is the rehydrated material mass, g; *m*_1_ is the rehydrated material before the material mass, g.

### 2.5. Color attributes

Fresh and dried sea buckthorn berries were selected. The fresh sea buckthorn berries were homogenized under ice bath conditions and then measured, and the dried sea buckthorn berries were ground into powder and passed through a 40-mesh sieve. The colorimetric L*, a*, and b* values were measured using SMY-2000SF colorimeter (Beijing Mingyang Technology Development Co., Ltd., Beijing, China). The total color difference value Δ*E* between the treated group and the fresh sample was also calculated according to Eq. 5 (23):


(5) $$\Delta \,E=\sqrt{{\left(L-L^{*}\right)}^{2}+{\left(a-a^{*}\right)}^{2}+{\left(b-b^{*}\right)}^{2}}$$

Where L*, *a*, and *b* are the brightness, red-green, and blue-yellow values of the sample before drying, respectively; and L*, a*, and b* are the brightness, red-green, and blue-yellow values of the sample after drying, respectively.

### 2.6. Non-enzymatic browning index

Non-enzymatic browning index analysis was performed according to a method of Deng et al. (16). The 2.0 g of sea buckthorn was first ground homogeneously with 20 mL of distilled water. The intermixture was centrifuged at 10,000 r/min for 30 min at 4°C. The browning degree represents the absorbance of the supernatant at the 420 nm (spectrophotometer Beijing Purkinje General Instrument Co., Ltd., Beijing, China), with the values calculated as a dry basis.

### 2.7. Texture analysis

The textural characteristics of sea buckthorn berries were determined using a TA.XT2 texture analyzer (Stable Micro System, UK) at 25°C (24). The test was performed using a cylindrical indenter of Φ36 mm with a pre-test speed of 1.0 mm/s, test speed of 0.5 mm/s, post-test speed of 1.0 mm/s, sample deformation of 50%, and a trigger induction force of 0.049 N. The hardness value is equal to the peak of the force in the curve, which is the maximum force (N) required to rupture the sample. The crispness result is expressed by the number of peaks generated during the test; the higher the number of peaks, the better the crispness of the product. The test was repeated 10 times, and the results were averaged.

### 2.8. Total flavonoids

Total flavonoid content (TFC) was determined according to the method of Zhang et al. (25). The absorbance was used as the vertical coordinate, and the mass concentration of the standard solution as the horizontal coordinate to draw the standard curve. The absorbance was used to calculate the mass concentration to obtain the regression equation.

Extraction of total flavonoids: 1.00 g of sea buckthorn berries was weighed accurately, and 20 mL of 95% ethanol solution was added in a material-liquid ratio of 1:20. The extract was sonicated at 50°C and 150 W for 40 min. The extract was obtained *via* extraction with a 0.45-μm organic filter membrane, and 1 mL of the extract was used for the determination. Absorbance was measured at 510 nm using a UV spectrophotometer. The total flavonoid content of the sample was expressed as mg rutin equivalent per gram (mg RE/g DW). Total flavonoid content was calculated according to Eq. 6:


(6) $$T\,o\,t\,a\,l\,f\,l\,a\,v\,a\,n\,o\,i\,d\,c\,o\,n\,t\,e\,n\,t=\frac{x\cdot V\cdot n}{m}$$

where *x* is the experimentally measured total flavonoid mass concentration/(mg/mL); *V* is the volume of the extraction solution/mL; *n* is the dilution multiple; and *m* is the dry basis mass of the sample (g).

### 2.9. Total phenols

Total phenol content (TPC) was determined according to the method of Ai et al. (26). Ten milligrams of gallic acid standard was weighed, dissolved in distilled water, and fixed to 100 mL to obtain a solution with a concentration of 100 mg/L. The solution was stored at 4°C in a refrigerator in the dark. The regression equation was obtained by plotting the standard curve. The extraction method for total phenols was the same as that for total flavonoids, and 1 mL of the extract was taken after extraction for determination (absorbance at 765 nm). The results were expressed as milligrams of equivalent gallic acid per gram of sample (mg GAE/g DW), and the total phenol content was calculated according to Eq. 7:


(7) $$T\,o\,t\,a\,l\,p\,h\,e\,n\,o\,l\,c\,o\,n\,t\,e\,n\,t=\frac{x\cdot V\cdot n}{m}$$

where *x* is the experimentally measured mass concentration of total phenols/(mg/mL); *V* is the volume of the extract/mL; *n* is the dilution multiple; and *m* is the dry basis mass of the sample (g).

### 2.10. Ascorbic acid

The ascorbic acid content in large-fruited sea buckthorn berries was determined using the titration method described by Deng at al. (16), with slight modifications. In total, 1.0 g of sea buckthorn powder was weighed and added to a mortar, followed by the addition of 20 mL of 20 g/L oxalic acid solution; thereafter, the mortar containing this mixture was placed in an ice bath and its contents were ground into a slurry. The slurry was then transferred to a 20-mL volumetric flask, fixed with 20 g/L oxalic acid solution, shaken well, and centrifuged at 8,000 *g* for 10 min. To avoid the color effect of the sample solution, the ascorbic acid content in the sample was calculated using the 2,6-dichlorophenol indophenol reverse titration method expressed as the dry basis (mg/100 g DW), which was calculated according to Eq. 8:


(8) $$A=\frac{c\cdot V_{1}\cdot V_{2}}{V_{3}\cdot W}$$

where A is the vitamin C content, mg/100 g DW; c is the concentration of L(+)-ascorbic acid standard solution, mg/mL; V1 is the volume of the ascorbic acid standard solution consumed for the titration of 5 mL of 2,6-dichloroindophenol sodium salt, mL; V2 is the total volume of the sample solution; V3 is the volume of the sample solution consumed for the titration of 5 mL of 2,6-dichloroindophenol sodium salt; and W is the dry weight of the sample taken (g).

### 2.11. Microstructure

Changes in the surface microstructure of sea buckthorn berries during drying were examined using scanning electron microscopy (JEOL, SU3500, Tokyo, Japan) (16). The dried sea buckthorn berries were cut into 5 mm × 5 mm slices with a razor blade, and the samples were fixed on a sample stage with double-sided tape and observed at a magnification of 500, an accelerating voltage of 5 kV.

### 2.12. Statistical analysis

Data on drying and quality tests were expressed as the mean ± standard deviation (SD) of three measurements and were analyzed using ANOVA and Duncan’s multiple range test using SPSS statistical software (version 21.0, Inc., USA). Statistical significance was set at *P* < 0.05.

## 3. Results and discussion

### 3.1. Drying characteristics

**FIGURE 3:**
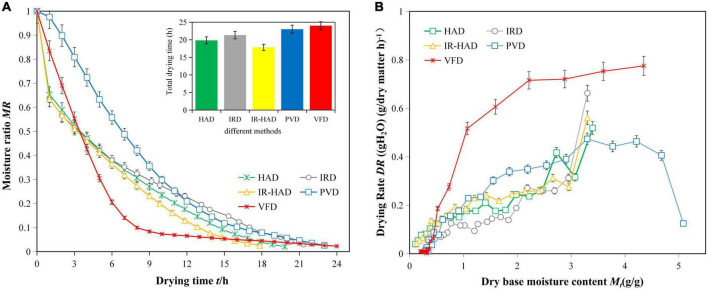
**(A,B)** The drying curves of sea buckthorn under different drying methods.

The average initial moisture content of sea buckthorn berries was found to be 83.32 ± 0.50%. Figures 3A, B shows the effect of different drying methods on the drying kinetics of sea buckthorn berries, and the different methods show significant differences in the drying kinetics. The drying times of HAD, IRD, IR-HAD, PVD, and VFD were 19.83, 21.33, 17.83, 23.00, and 24.00 h, respectively. IR-HAD took the shortest time, VFD took the longest time, and the drying time of PVD was 15.99, 7.83, and 29.00% longer than HAD, IRD, and IR-HAD, respectively; the drying time of VFD was 21.03, 12.52, and 34.60% longer than HAD, IRD, and IR-HAD, respectively.

In the drying process, PVD dries the material through pressure change, HAD dries the material by eliminating surface moisture (16), IRD dries the material by radiation penetration (12), and IR-HAD dries the material by the combined action of both (16). Thus, IR-HAD can transfer more moisture and has a shorter drying time, making it the most efficient method.

Based on the drying rate variation curve, the drying rates of the different drying methods varied, and there was a large difference from the beginning of drying; however, the variation curves of HAD, IRD, and IR-HAD were essentially similar, unlike those of VFD and PVD. There are three drying phases in PVD, namely preheating, constant rate, and decreasing periods, which is consistent with the results of Liu et al. (21), and Deng et al. (16). The drying rates of HAD, IRD, IR-HAD, and VFD decreased with decreasing moisture content, and the entire stage of drying occurred in the decreasing drying period, indicating that moisture diffusion affects the drying process.

Compared with IR-HAD and HAD, IRD dried the samples faster in the early stages, but the total drying time was longer, which may be related to the stronger penetration of IR radiation, which generates heat quickly and increases the rate of moisture removal (17). In HAD, the gas flow rate is rapid, and the boundary layer between material surface and air is thin; therefore, HAD has a high convective heat transfer coefficient. However, the high air flow and convective heat transfer coefficient can lead to rapid dehydration on the surface of sea buckthorn, causing rapid shrinkage at the early stage of drying and rendering it difficult to diffuse moisture outward inside the berry. In IR-HAD, infrared radiation is absorbed and converted to heat on the material surface. Consequently, the moisture transfer effect of IR-HAD is enhanced; however, at the same time, the high air flow rate during IR-HAD dissipates the heat from the material surface. In the late stage of drying, the combined water present in sea buckthorn is difficult to remove by heat conduction; however, the heat generated by IR radiation during the heating process can penetrate the material and combine with the moisture, which is beneficial for the internal water vapor pressure and moisture removal. Moreover, the drying rate of PVD was higher than that of HAD, IRD, and IR-HAD in the early stages, but the overall drying time was longer. This may be due to the room-temperature cold air entering the drying chamber in the atmospheric pressure state of PVD, which requires re-heating, and a large amount of heat is withdrawn during the operation of the vacuum pump, prolonging the drying time (16). VFD and PVD had significantly lower rates in the later stages of drying, which may be because the intracellular bound water was more difficult to remove by alternating cycles of freezing and vacuum atmospheric pressure, thus prolonging the drying time.

### 3.2. Rehydration ratio

**FIGURE 4:**
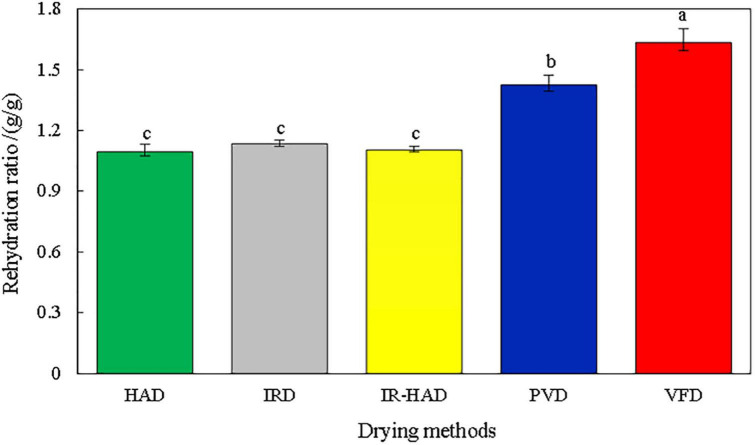
Effect of different drying methods on rehydration ratio of sea buckthorn berries. Different letters in the figure reveal significant differences (*P* < 0.05) according to the Duncan test.

The rehydration ratio is an important indicator in evaluating the dried product and can characterize the degree of destruction of the material structure due to drying (22). A higher rehydration ratio corresponds to better product quality, indicating minimal damage to the product structure (16). Figure 4 shows the effect of the different drying methods on the complex water ratio of dried sea buckthorn berries, which is closely related to changes in the microstructure, thus determining the macroscopic properties (21).

In this study, changes in the microstructure of sea buckthorn berries had a significant effect on the rehydration capacity, with significant differences between most of the groups. No significant differences were observed for HAD, IRD, and IR-HAD. VFD-dried berries had the best rehydration rate. The vacuum and freezing environment prevented the possibility of structural destruction of sea buckthorn berries, forming a stable solid skeleton that ensured the original structure of the berries; thus, the porous structure caused by VFD gave it a high rehydration rate. It was found that PVD increased the formation of “tunnels” during the drying process and enhanced the water transfer. The experimental results confirmed those reported by Liu et al. (21) and Wang et al. (27), who found that PVD produced a tunneling effect that enlarged and interconnected the material micropores. For HAD, IRD, and IR-HAD, the drying time was approximately the same. With these three methods, the hot air and IR radiation may have caused the structural collapse of the internal matrix of sea buckthorn berries, which could have prevented the water absorption and lowered the rehydration capacity.

### 3.3. Color

Color is an important attribute in evaluating the merit of food products, affecting the consumer’s choice and value of the product (28). Table 1 shows the effects of the different drying methods on the color of sea buckthorn berries (L*, a*, b*, and Δ*E*). The brightness values of fresh sea buckthorn berries decreased from 53.44 ± 0.30 to 36.00 ± 0.12, which may be attributed to the reduction in brightness due to the Maillard reaction owing to drying and oxidation. The brightness values of VFD-dried berries were the highest among the different drying methods at 44.18 ± 0.19. This was due to the vacuum environment inhibiting enzymatic browning and the occurrence of the Maillard reaction, thus ensuring the original color of sea buckthorn berries; consistent with the study of Liu et al. (21). The second highest brightness value was observed for PVD-dried berries (42.60 ± 0.29), which was significantly higher than that of HAD-, IRD-, and IR-HAD-dried berries. This may be because the vacuum environment prevents the occurrence of enzymatic browning and oxidation reactions, consistent with the findings of Deng et al. (16). The higher L* of infrared-dried berries than that of hot-air-and infrared-assisted hot-air-dried berries may be attributed to uneven temperature distribution on the surface of the sea buckthorn berries during heating. Localized overheating accelerating the browning of the surface of sea buckthorn berries also led to an uneven distribution of internal moisture, which affected the drying rate. It may also lead to the surface crusting phenomenon in the late stage of drying, where the internal structure does not reach a safe moisture content, thus promoting non-enzymatic browning and related biochemical reactions and resulting in color deterioration.

**TABLE 1:** The color parameters of sea buckthorn under different drying methods.

Parameter	Fresh	Drying methods				
		**HAD**	**IRD**	**IR-HAD**	**PVD**	**VFD**
	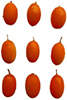	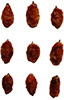	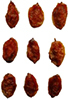	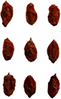	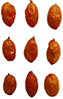	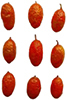
L*	53.44 ± 0.30^a^	36.39 ± 0.45^e^	37.58 ± 0.19^d^	36.00 ± 0.12^f^	42.60 ± 0.29^c^	44.18 ± 0.19^b^
a*	25.35 ± 0.13^c^	22.05 ± 0.89^e^	24.31 ± 0.21^d^	22.11 ± 0.59^e^	29.73 ± 0.41^b^	33.25 ± 0.20^a^
b*	47.42 ± 0.29^a^	30.96 ± 1.84^e^	35.51 ± 0.25^d^	32.52 ± 1.05^e^	42.34 ± 0.83^c^	44.33 ± 0.43^b^
Δ*E*	*	23.95 ± 1.50^a^	19.86 ± 0.28^b^	22.43 ± 0.88^a^	12.76 ± 0.42^c^	12.56 ± 0.13^c^
Browning index /(Abs/g d.m.)	0.09 ± 0.00^f^	0.42 ± 0.01^b^	0.35 ± 0.01^c^	0.59 ± 0.01^a^	0.28 ± 0.00^d^	0.24 ± 0.00^e^

Different letters in the figure reveal significant differences (*P* < 0.05) according to the Duncan test.

Table 1 shows that the b* values of all dried sea buckthorn berry samples were significantly (*P* < 0.05) lower than those of fresh samples, which can be explained by the degradation of flavonoids in sea buckthorn berries (4). VFD- and PVD-dried berries retained the product yellowness to a greater extent than those dried using HAD, IRD, and IR-HAD. This may be because VFD and PVD can minimize the possibility of oxidation of flavonoids in an anoxic environment (16, 21), as oxidation reaction is the main cause of flavonoid loss (12). While the a* of VFD- and PVD-dried berries was significantly higher than that of fresh sea buckthorn berries, which may be because drying promotes carotenoid production, the low-oxygen environment promotes the formation of some compounds, resulting in the red coloration of sea buckthorn berries. This is consistent with the results of Jiang et al. (29) in their study on the drying of *Panax ginseng* roots.

Bondaruk et al. (30) found that color changes could be observed by the naked eye with Δ*E* values greater than five. The Δ*E* was greater than 12 for sea buckthorn berries with different drying methods compared to fresh sea buckthorn berries, indicating that drying significantly changed the color of sea buckthorn berries. The color changes were mainly due to changes in L* and b*. VFD- and PVD-dried berries had the smallest Δ*E* values of 12.56 ± 0.13 and 12.76 ± 0.42, respectively. The dried fruits were brightly colored and showed bright yellow color, which was significantly lower than those dried using the other three drying methods (*P* < 0.05), indicating that VFD and PVD were able better to maintain the color of sea buckthorn berries.

### 3.4. Browning index

The browning index is usually used to evaluate the degree of enzymatic browning (26). Table 1 shows the browning index variations for the different drying methods. VFD-dried berries had the lowest browning index (0.24 Abs/g d.m.), followed by PVD- (0.28 Abs/g d.m.), IRD- (0.35 Abs/g d.m.), HAD- (0.42 Abs/g d.m.), and IR-HAD–dried berries (0.59 Abs/g d.m.). This may be due to the vacuum environment of VFD and PVD preventing the occurrence of the Maillard reaction (31). The contact with oxygen during the drying by HAD, IRD, and IR-HAD may have led to enzymatic browning, probably due to the synthesis of polyphenol oxidase during drying (16). The data showed a negative correlation between the browning index and L*, which is consistent with the results of Deng et al. (16).

### 3.5. Texture properties

The final moisture content of sea buckthorn berries varied among the different drying methods and affected the texture of the dried material: the lower the moisture content, the higher the hardness and brittleness of sea buckthorn berries (24). The results are presented in Table 2. The HAD group exhibited the highest hardness, potentially because the hot air quickly took away the moisture from the surface of the material. This caused the crusting phenomenon, making it difficult to transfer moisture to the surface during the later stages of drying, ultimately forming a hard dry film (32). Meanwhile, HAD, IRD, and IR-HAD were more destructive to the cellular tissue structure of sea buckthorn berries, with a more compact overall structure, thus presenting higher hardness values. VFD maintains the original structure of the material’s tissue cells and causes less shrinking, so the texture of the dried sea buckthorn berries was softer and more flimsy. This, however, makes it difficult to recover the pre-deformation degree after being compressed, therefore yielding the lowest reversibility. PVD elicits a hollow and porous structure with low overall shrinkage of the material; the hardness and brittleness, therefore, were the lowest.

**TABLE 2:** Effects of different drying methods on the texture of sea buckthorn.

Drying methods	Hardness /N	Fracturability /N	Adhesiveness/g⋅s^–1^	Springiness	Cohesiveness	Gumminess	Chewiness	Resilience
HAD	71.88 ± 5.15^a^	37.14 ± 5.59^a^	−0.68 ± 0.58^a^	0.48 ± 0.02^a^	0.30 ± 0.05^a^	21.38 ± 3.53^a^	10.20 ± 1.69^a^	0.13 ± 0.03^a^
IRD	51.94 ± 2.86^b^	29.52 ± 4.39^a,b^	−0.48 ± 0.08^a^	0.53 ± 0.01^a^	0.28 ± 0.03^a^	14.68 ± 1.03^b^	7.84 ± 0.72^b^	0.10 ± 0.01^b^
IR-HAD	26.43 ± 0.84^c^	25.13 ± 3.17^b^	−0.78 ± 0.19^a^	0.47 ± 0.05^a^	0.17 ± 0.01^b^	4.39 ± 0.39^c^	2.02 ± 0.03^c^	0.07 ± 0.00^b^
PVD	3.55 ± 0.18^e^	3.76 ± 0.04^c^	−1.08 ± 0.46^a^	0.51 ± 0.22^a^	0.08 ± 0.01^c^	0.30 ± 0.04^d^	0.16 ± 0.08^d^	0.02 ± 0.00^c^
VFD	9.12 ± 0.29^d^	*	−1.93 ± 1.19^a^	0.51 ± 0.02^a^	0.34 ± 0.05^a^	3.12 ± 0.21^c,d^	1.58 ± 0.20^c,d^	0.04 ± 0.00^c^

Different letters in the figure reveal significant differences (*P* < 0.05) according to the Duncan test.

Chewiness is a reflection of the energy required to move the food from a chewable state to a swallowable state, which integrates the sustained resistance of the sample to chewing. Its value is the product of hardness, cohesiveness, and elasticity, thus having a positive correlation with hardness variation (33). HAD-dried berries had the highest chewiness, which may be due to the surface heating of the drying process, which led to a significant increase in the chewiness of the sea buckthorn berries. The adhesiveness also showed significant differences between the different drying methods, which may be due to the different levels of reducing and total sugars in differently dried sea buckthorn berries (34). Thus, PVD and VFD could better retain reducing sugar substances in sea buckthorn berries, while IRD-dried berries exhibited lower adhesion due to the penetration of thermal radiation that destroyed reducing sugars and other substances.

### 3.6. TFC and TPC

**FIGURE 5:**
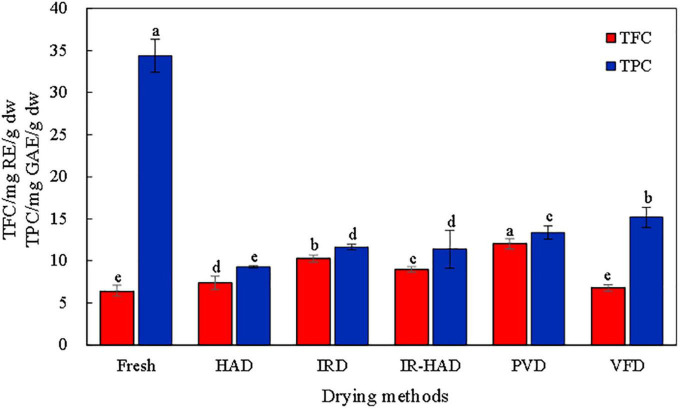
Effects of different drying methods on total flavonoid content (TFC) and total phenolic content (TPC) of sea buckthorn berries. Different letters in the figure reveal significant differences (*P* < 0.05) according to the Duncan test.

Different drying methods showed significant differences in the total flavonoid content of sea buckthorn berries. The results are presented in Figure 5. It was found that the flavonoid content increased after drying, but after VFD, the flavonoid content of the berries did not differ significantly from that of fresh sea buckthorn berries, which may be due to the high temperature stress during the drying process, which activates the entire system to resist the external environmental changes (35). It is also possible that the high temperatures disrupted the cell wall structure of sea buckthorn berries and reduced the polyphenol oxidase and peroxidase activities, allowing more flavonoid compounds to be released (36), or that the free flavonoids in fresh sea buckthorn berries were converted to flavonoids by condensation reactions during drying, thus leading to an increase in flavonoid content (37). We also found that sea buckthorn berries had the highest flavonoid content after PVD, which could be due to the alternating cycles of pressure that led to the rupture of the cell structure and the release of more flavonoids. IRD penetration also had high flavonoid content, possibly due to a similar mechanism.

Unlike total flavonoids, fresh sea buckthorn berries had the highest total phenolic content, which was significantly higher than that of dried sea buckthorn berries. VFD and PVD helped to retain the phenolics in the sea buckthorn berries after drying. VFD reduces the water content through sublimation, while the vacuum environment avoided contact with oxygen, which reduced the oxidative degradation of phenolics. The vacuum environment in PVD also helped avoid the oxidation reaction. HAD not only had a longer drying time but also had a long time in contact with oxygen in the air; thus, the loss of phenolic substances was the most severe.

### 3.7. Ascorbic acid

**FIGURE 6:**
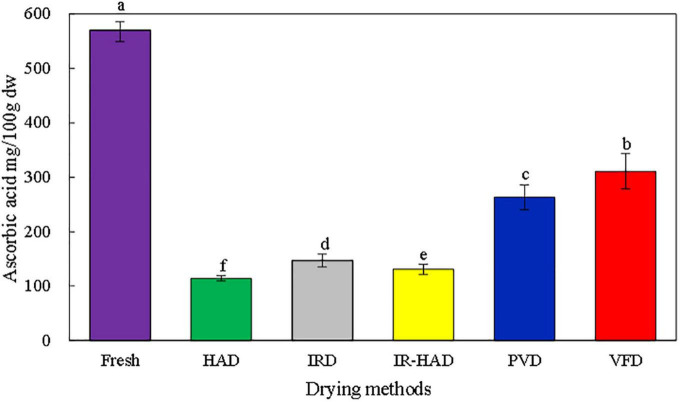
Effects of different drying methods on ascorbic acid content of sea buckthorn berries. Different letters in the figure reveal significant differences (*P* < 0.05) according to the Duncan test.

The content of ascorbic acid in fresh sea buckthorn berries was 570 mg/100 g. Figure 6 shows that the ascorbic acid content of sea buckthorn berries decreased by 45.39, 53.81, 74.23, 77.09, and 79.93% after VFD, PVD, IRD, IR-HAD, and HAD, respectively, compared to the fresh samples. The retention of ascorbic acid was higher in the VFD samples at 311.27 mg/100 g, mainly because VFD avoids heat and oxidation in sea buckthorn berries, thus reducing the degradation of ascorbic acid, which is consistent with a study on VFD on cornelian cherries by Silva-Espinoza et al. (15). While the vacuum environment of PVD also limited oxidation and reduced the possibility of ascorbic acid degradation, the higher drying temperature of HAD, IRD, and IR-HAD resulted in more heat-related loss of ascorbic acid content in the material.

### 3.8. Microstructure

**FIGURE 7:**
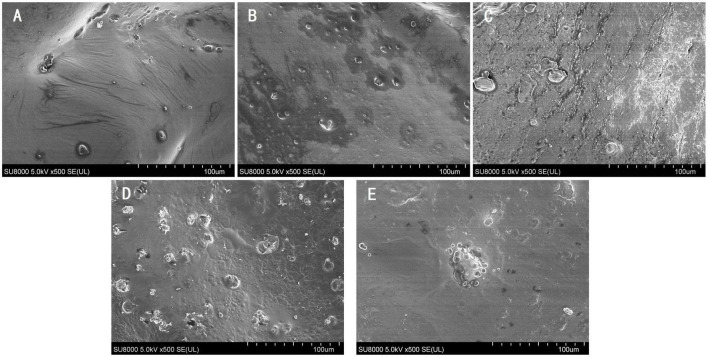
Microstructure observation (500 × magnifications) of sea buckthorn berries under different drying methods. **(A)** Hot air drying (HAD); **(B)** infrared drying (IRD); **(C)** infrared-assisted hot air-drying (IR-HAD); **(D)** pulsed vacuum drying (PVD); **(E)** vacuum freeze drying (VFD).

Studies have shown that microstructure affects the performance and quality of dried products; therefore, understanding microstructure can help to resolve the mechanism of material quality changes at the cellular level and improve drying performance (38, 39). To investigate the effect of the different drying methods on the microstructure of sea buckthorn berries, scanning electron microscopy images of the dried berries were selected, as shown in Figure 7. The surface of HAD berries (Figure 7A) was partially folded, which may be due to the folding of the epidermis because of the rapid evaporation of surface water due to hot air. The IRD surface was smoother and had some holes, which might be due to the stronger penetration of IR radiation that destroyed the cellular structure on the surface of sea buckthorn berries, while the shadows around the holes might have been generated by the diffusion of internal oil-like substances to the outside of the cells. In contrast to HAD- and IRD-dried berries, the surface of IR-HAD-dried berries not only had pores but also microcracks, which might be caused by the combined effect of air speed and IR radiation, promoting the diffusion of water from the interior of the cell to the outside. Many pores were observed on the surface of sea buckthorn berries in the PVD samples, which was also observed by Liu et al. (21). This phenomenon can be explained by the large pressure difference between the inside and outside of the material due to the vacuum illusion that lowered the boiling point of water in sea buckthorn berries. The alternating cycles of vacuum and atmospheric pressure lead to the continuous expansion of the pores, which also explains why the same pore shape is produced, but there is no shadow around the PVD pores like the one produced by the diffusion of greasy substances after IRD treatment. The higher rehydration capacity of sea buckthorn berries after PVD treatment also supports this conjecture. The VFD samples had a smooth surface and no extravasation of grease, which may be due to the direct sublimation of water from a solid to a gaseous state, effectively ensuring the original structural properties of the material.

## 4. Conclusion

This study investigated the effects of five different drying methods, namely HAD, IRD, IR-HAD, PVD, and VFD, on the drying kinetics, physicochemical properties, and microstructure of sea buckthorn berries. The results showed that the different drying methods had considerable different effects on the drying characteristics, color, total phenols, total flavonoids, rehydration ratio, and texture of sea buckthorn berries. IR-HAD had the shortest drying time, and VFD had the longest time. PVD reduced the occurrence of non-enzymatic browning and better preserved the surface of sea buckthorn berries. VFD and PVD elicited the brightest color and lowest browning in dried sea buckthorn berries, as well as the highest retention of total phenols and ascorbic acid. Sea buckthorn berries obtained by VFD had the best quality, but the long drying time and high cost of VFD hinder its potential for industrial application. PVD, on the other hand, has a low cost and large batch size and could, therefore, be employed industrially to ensure the maximum drying quality while reducing the drying time and energy consumption.

## Data availability statement

The original contributions presented in this study are included in the article/supplementary material, further inquiries can be directed to the corresponding author.

## Author contributions

ZG: conceptualization, methodology, and writing—original draft preparation. LZ: formal analysis investigation and resources. JW and XLY: methodology and software. ML and WY: formal analysis investigation. BH and QZ: investigation. XHY: resources, validation, funding acquisition, supervision, and writing—reviewing and editing. All authors contributed to the article and approved the submitted version.
